# The interplay of thyroid hormones and the immune system – where we stand and why we need to know about it

**DOI:** 10.1530/EJE-21-1171

**Published:** 2022-02-17

**Authors:** Christina Wenzek, Anita Boelen, Astrid M Westendorf, Daniel R Engel, Lars C Moeller, Dagmar Führer

**Affiliations:** 1 Department of Endocrinology, Diabetology and Metabolism, University Hospital Essen, University Duisburg-Essen, Essen, Germany; 2 Endocrine Laboratory, Department of Clinical Chemistry, Amsterdam Gastroenterology Endocrinology and Metabolism, Amsterdam UMC, University of Amsterdam, Amsterdam, Netherlands; 3 Institute for Medical Microbiology, University Hospital Essen, University Duisburg-Essen, Essen, Germany; 4 Institute for Experimental Immunology and Imaging, University Hospital Essen, University Duisburg-Essen, Essen, Germany

## Abstract

Over the past few years, growing evidence suggests direct crosstalk between thyroid hormones (THs) and the immune system. Components of the immune system were proposed to interfere with the central regulation of systemic TH levels. Conversely, THs regulate innate and adaptive immune responses as immune cells are direct target cells of THs. Accordingly, they express different components of local TH action, such as TH transporters or receptors, but our picture of the interplay between THs and the immune system is still incomplete. This review provides a critical overview of current knowledge regarding the interaction of THs and the immune system with the main focus on local TH action within major innate and adaptive immune cell subsets. Thereby, this review aims to highlight open issues which might help to infer the clinical relevance of THs in host defence in the context of different types of diseases such as infection, ischemic organ injury or cancer.

## Introduction

Thyroid hormones (THs) are critical regulators of various physiological processes within the human body, which become particularly evident in case of imbalance. Both deficiency and excess of THs are associated with severe disorders affecting different organs. Interestingly, hypothyroidism has been associated with increased susceptibility to infectious diseases. Initial studies in the 1970s and 1980s suggested that THs directly act on human leukocytes during bacterial pneumonia and promote leukocyte function given an increased phagocytic activity of neutrophils and augmented lymphocyte proliferation upon TH administration *in vivo* (1, 2). Moreover, hypothyroidism was identified as a risk factor for periprosthetic joint infections based on a meta-analysis of institutional databases on patients with arthroplasty (3). In line with this, increased mortality of hypothyroid rats was observed in a caecal ligation and puncture model of sepsis (4). Over the past years, evidence for a direct impact of THs on the immune system is growing. Specifically, different TH transporters (THTs), deiodinases (DIOs) and TH receptors (THRs) were shown to be expressed within immune cells (5, 6). Yet, a comprehensive understanding about the local control of TH action within the different components of the immune system is still missing. Furthermore, data on how THs impact the function of different immune cells are incomplete or controversial. For example, increased production of reactive oxygen species (ROS) by neutrophils was observed during subclinical hypothyroidism and upon *in vitro* stimulation of neutrophils with THs, indicating stimulatory and inhibitory actions of THs within the same immune cell (7, 8).

This review will provide an overview of current concepts and knowledge regarding the interplay of THs and the immune system. To this end, the review briefly discusses changes of systemic TH homeostasis induced by the immune system and addresses in detail the local action of THs within major immune cell subsets including resident and recruited cells in the course of inflammation/infection. With this, we aim to highlight open issues, which need to be addressed for a better understanding of TH action during inflammatory and infectious diseases and ultimately to harness local TH action to open up new avenues for diagnosis, prognosis and treatment.

## Local action of THs

**Figure 1:**
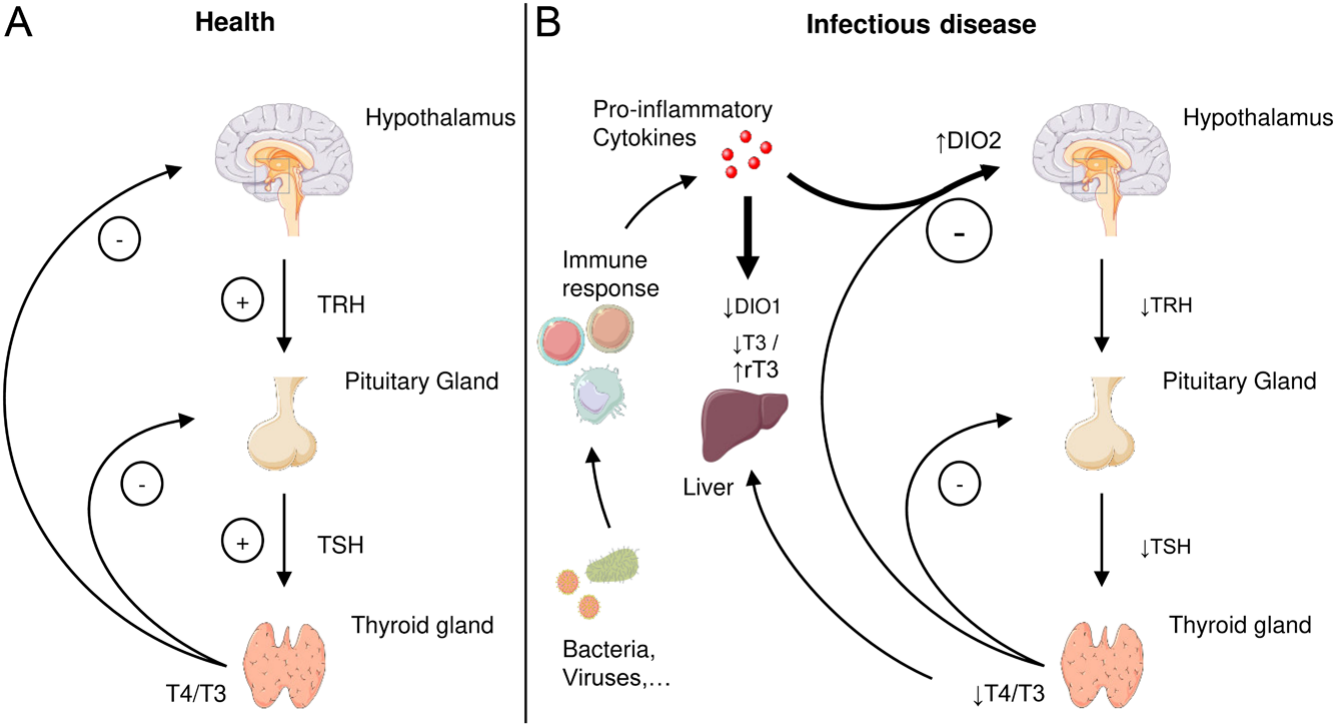
Schematic view of the hypothalamic–pituitary–thyroid (HPT) axis during health and infectious disease. (A) At steady-state thyroliberin (TRH) released from the hypothalamus stimulates the secretion of thyroid-stimulating hormone (TSH) by the pituitary gland which in turn drives thyroxine (T4) and triiodothyronine (T3) secretion from the thyroid gland. The resulting thyroid hormones have negative feedback on the hypothalamus as well as the pituitary gland. (B) During infectious disease pro-inflammatory cytokines locally augment the negative feedback of thyroid hormones (TH) on the hypothalamus via elevated deiodinase 2 (DIO2) expression and thereby diminish TRH and TSH secretion. Moreover, inflammatory stimuli shape peripheral TH metabolism limiting DIO1 expression in the liver. As a result, reduced serum T3 and /or T4 levels are observed during severe illness whereas reverse T3 (rT3) concentrations may be elevated.

The secretion of THs by the thyroid gland is controlled at two levels of the hypothalamus-pituitary-thyroid (HPT) axis (for review see (9)). In short, thyrotropin-releasing hormone (TRH) is secreted by the hypothalamus stimulating the release of the thyroid-stimulating hormone (TSH) from the pituitary which in turn drives the production and secretion of THs by the thyroid gland. Homeostasis of systemic TH levels is achieved by negative feedback to the hypothalamus and the pituitary (Fig. 1). Interestingly, the human thyroid gland mainly secretes thyroxine (T4), whereas triiodothyronine (T3) is mostly generated by deiodination in peripheral tissues (10, 11). On the contrary, in rodents, which are commonly used in basic endocrine research, the thyroid gland secrets significant amounts of T3 underlining the relevance of species-specific differences in TH synthesis and metabolism (12, 13).

**Figure 2:**
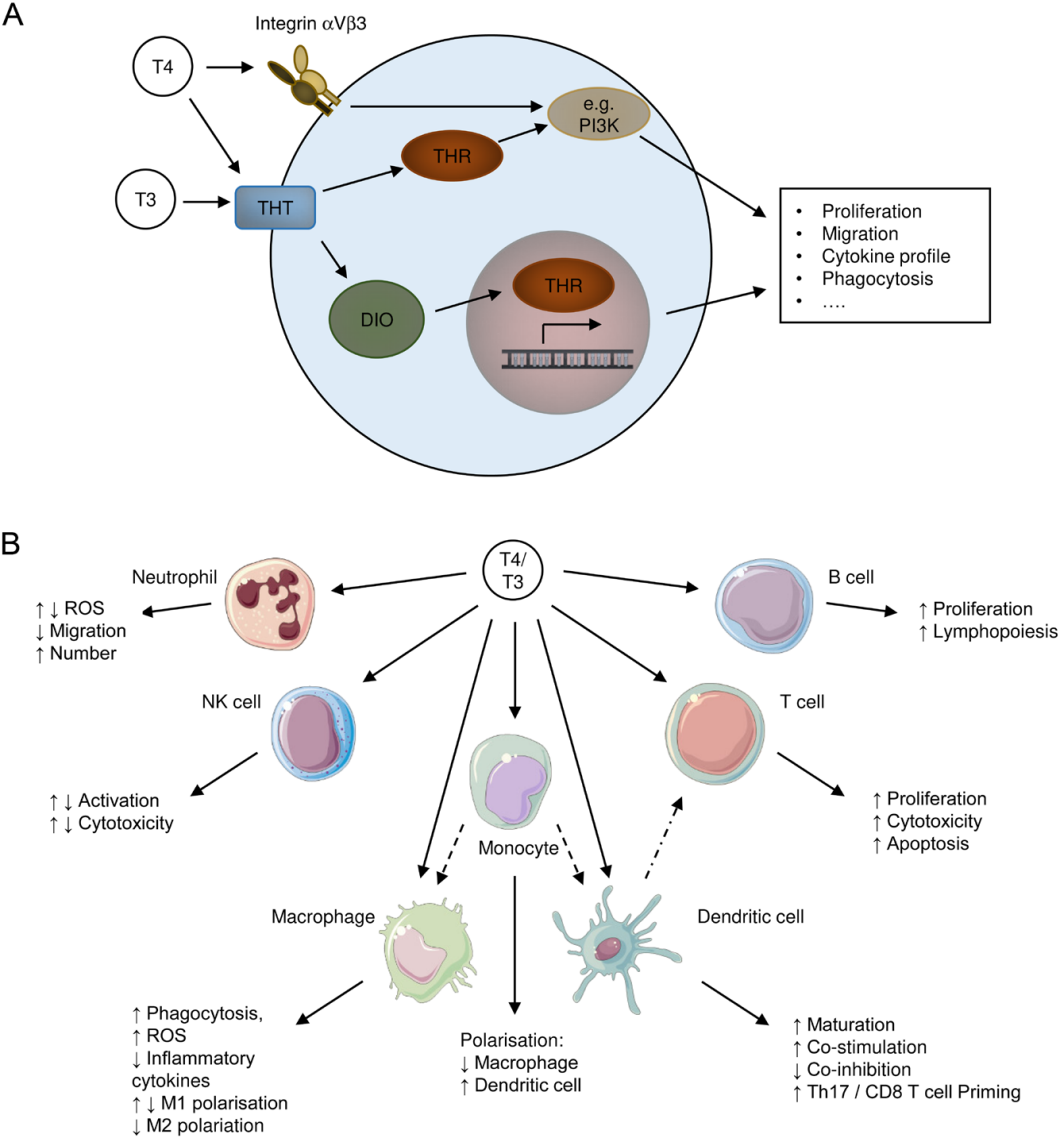
Local action of thyroid hormones in the immune system. (A) Various immune cells were described to express different types of thyroid hormone transporters (THTs) facilitating the uptake of THs into the cell. Within the cell deiodinases (DIOs) convert THs either promoting or limiting TH activation. Intracellular T3 can subsequently bind to TH receptors (THRs) in the cytoplasm or the nucleus initiating noncanonical or canonical signalling pathways, respectively. In addition to noncanonical THR action, which relies among others on PI3K signalling pathways, T4 can bind to integrin αVβ3 on the cell surface initiating several pathways such as PI3K signalling. (B) Local action of THs was detected in different innate and adaptive immune cells such as neutrophils, natural killer (NK) cells, macrophages, monocytes, dendritic cells, T cells and B cells. Here, T3 and T4 were described to directly regulate various functional aspects including activation, differentiation, proliferation and/or migration. Moreover, TH signalling in monocytes and dendritic cells indirectly affects macrophage and dendritic cell responses as well as T cell activity, respectively.

Although in the classical view, TH action in the body is largely determined by circulating TH concentrations, the concept of local action of THs at the tissue- or cell-specific level has gained increasing interest in recent years. Local action of THs depends on three steps: (i) TH transport into the target cell; (ii) TH metabolism into active or inactive hormone; (iii) TH binding to THRs, which mediate TH signalling by canonical or noncanonical modes of action. These distinct aspects of local TH action may occur in a tissue and cell-specific manner and vary in health and disease leading to an overall highly dynamic modulation of TH effects in an organism (Fig. 2).

### TH transporter

For a long time, transmembrane transport of THs has been assumed to occur via passive diffusion. However, since 2004 several THTs were identified that actively mediate TH uptake from a cell (for review see (14)). Of these transporters, monocarboxylate transporter 8 (MCT8) is best-known as it is highly specific for TH transport and inactivating mutations of MCT8 result in the X-linked Allan-Herndon-Dudley Syndrome in humans. Other known THTs include MCT10, large neutral amino acid transporters (LATs) and organic anion transporter polypeptides (OATPs). Cellular availability of THs is regulated by the cell’s equipment with THTs and their specific TH transport characteristics. For example, MCT8 can transport T4 and T3 with similar efficiency, whereas OATP4A1 preferentially transports T3 into target cells and LAT2 displays higher affinities for the TH metabolite 3,3’ T2 (15, 16, 17, 18). In addition to TH influx, THTs also control the efflux of THs from cells with MCT8 exerting a prominent role in TH secretion from the thyroid gland (14, 19).

### TH deiodinases

Within the target tissue/cell, local TH concentrations are further adjusted by different DIOs (for review see (20)). Three different DIOs are known, of which DIO1 and DIO2 can catalyse the conversion of T4 into T3 with DIO2 being more efficient (21). Inactivation of THs is mediated by DIO1 and DIO3 converting T4 and T3 into reverse-T3 (rT3) and 3,3’-T2, respectively. Similar to THTs, DIOs display different affinities for THs (21, 22, 23), and interestingly, THs may directly regulate the expression of DIOs. For example, T3 drives the expression of DIO1 and DIO3 whereas T3 and T4 downregulate DIO2 mRNA expression and activity, respectively (24, 25, 26, 27, 28).

### TH receptors

Signalling of THs within the cell is mediated by different THRs, which act as ligand-modulated transcription factors and exert a dual mode of action (for review see (29)). THRs are linked to TH response elements and binding of T3 induces a switch in transcription factor activity for positive and negative TH target genes based on a ligand-triggered replacement of corepressors and coactivators, respectively. Three main T3-binding THR isoforms have been identified to date, THRa1, THRb1 and THRb2. In addition to this classical concept of canonical mode of action, THRs may additionally act via noncanonical signalling (30, 31, 32, 33, 34, 35). Here, activation of rapid cytosolic signalling cascades, for instance phosphatidylinositol-3-kinase (PI3K) or extracellular-signal-regulated kinase (ERK) pathways, are mediated by ligand-bound THRs. Independent of THRs, THs can also interact with integrin αVβ3 (vitronectin receptor) which was found to be expressed on the cell surface of different cells such as endothelial cells or T cell lymphomas (29, 36). The binding of THs to integrin αVβ3 was shown to initiate signalling via PI3K or ERK, however integrin αVβ3 does not solely bind THs (29, 37). Both, canonical and noncanonical TH signalling cascades are associated with various cellular processes such as proliferation, metabolism or migration.

## Interplay of THs and the immune system

The immune system facilitates the defence against a wide range of microorganisms facing the human body. Initially, the innate immune compartment affords a fast, albeit unspecific response, while later adaptive immunity takes over host defence, providing a slow though specific response. Several types of immune cells can be allocated either to the innate or the adaptive immune compartment, differentially facilitating pathogen recognition, elimination and immunological memory. However, excessive immune responses can be seriously deleterious and thus immunity needs to be tightly regulated.

The interplay of THs and the immune system involves a bidirectional crosstalk. On the one hand, pathological conditions related to severe illness or autoimmunity interfere with TH homeostasis. On the other hand, THs were depicted as regulators of innate and adaptive immune cells (2, 8).

### Non-thyroidal illness syndrome

During severe illness, for example, related to surgery or infection, serum T4 and T3 concentrations decrease despite normal or reduced TSH levels (38). Low T4 levels correlate with disease severity and are associated with increased mortality (39, 40). Although these hormonal changes resemble those observed in central hypothyroidism, the decline of circulating TH levels is transient as serum TH concentrations restore when the patients recover. Moreover, unlike central hypothyroidism, rT3 serum concentrations are frequently elevated in critically ill patients. Hence, this condition is referred to as non-thyroidal illness syndrome (NTIS) (38, 39).

Despite vast literature, the mechanisms of NTIS are not fully understood, but one main feature is the central downregulation of the HPT axis via TRH which leads to reduced TSH and TH levels as demonstrated in a lipopolysaccharide (LPS)-induced NTIS model (41, 42). Furthermore, alterations in TH metabolism due to a decreased expression of DIO1 in the liver are thought to account for reduced T3 and elevated rT3 levels (43). Interestingly, attenuated TRH expression during severe disease has been associated with increased DIO2 activity in the hypothalamus due to NFκB activation as a result of inflammatory stimuli ((42, 44), Fig. 1). Likewise, inflammatory cytokines were proposed to attenuate DIO1 expression in the liver during critical illness (45, 46). Different cytokines, for example, interleukin-1β (IL1β), IL6 or TNFα, were shown to correlate with diminished TH levels during NTIS (38, 47, 48), however blockade of a single cytokine such as IL1β or TNFα failed to identify a distinct cytokine as the primary driver of NTIS pathology.

Interestingly, besides the role of immune responses during NTIS pathology, the immune system per se might exert an important function in recovery (38). The secretion of TSH by immune cells has been proposed to support the restoration of TH homeostasis; however, the role of TSH in the immune system will be discussed in detail later on.

### Autoimmune thyroid disease

In addition to NTIS and much more frequent in the general population, autoimmunity can lead to thyroid gland dysfunction which may present as either primary hypo- or hyperthyroidism. Since a detailed discussion of these pathological conditions would go beyond the scope of this review, we only briefly highlight some core aspects here (for review see (49)).

Autoimmune thyroiditis is the major cause of non-surgical hypothyroidism and is characterised by infiltration of thyroid tissue with autoreactive T lymphocytes and measurable autoantibodies against two main thyroid gland proteins, thyroid peroxidase and thyroglobulin. As a result, of mostly chronic cellular immune processes, thyroid cells are destroyed, which over time may result in the critical reduction of functional thyroid tissue and thus hypothyroidism (49). In contrast, autoimmune hyperthyroidism, that is Graves’ disease, is characterised by autoreactive B cells secreting autoantibodies against the TSH receptor (TSHR). These biologically active antibodies mostly cause TSHR stimulation resulting in excessive TH production and secretion with a much more clinical manifestation than autoimmune thyroiditis (49). Typically, Graves’ disease patients exhibit a spectrum of different TSHR antibodies, in rare cases also with receptor blocking properties, which may change in the course of the disease and in sum determine thyroid gland activity.

The development of autoimmune thyroid diseases has been attributed to multiple intrinsic and extrinsic factors, with genetics playing a major role in disease susceptibility. In particular, several polymorphisms have been identified prominently in genes related to co-stimulation of immune cells (e.g. CD40), antigen presentation (e.g. HLA-DRβ-Arg74), or immune tolerance (e.g. FOXP3) (50).

In summary, these findings in NTIS and autoimmune thyroid diseases illustrate that the immune system has a crucial impact on TH homeostasis affecting the HPT axis and the thyroid gland itself.

## Local action of THs in innate immune cells

### Macrophages

Tissue-resident macrophages are among the first cells to respond to invading pathogens or inflammatory stimuli and can be found in various tissues (51). Upon pathogen encounter, tissue-resident macrophages are classically described to adopt either a pro-inflammatory M1 or anti-inflammatory M2 phenotype (52). In short, M1 macrophages facilitate the clearance of foreign microbes via phagocytosis and the generation of ROS, while M2 macrophages are involved in tissue repair (53). Both, M1 and M2 macrophages can be seen as the extremes of a spectrum of phenotypes. Moreover, tissue-resident macrophages orchestrate subsequent immune responses via the secretion of cytokines (e.g. IL1β, IL6 and TNFα) and chemokines (e.g. CCL2, CXCL1 and CXCL10) attracting further immune cells to the site of infection (51, 52).

Importantly, several proteins involved in local TH action were identified in macrophages (Table 1). For example, MCT10 expression was detected in the RAW264.7 murine macrophage cell line via real-time quantitative PCR (qRT-PCR) (54). Additionally, OATP4a1 and LAT2 mRNA and protein expression was demonstrated in tissue-resident macrophages in the brain (55). Based on qRT-PCR analyses, DIO2 was assumed to be related to intracellular TH metabolism in murine macrophages, and both THRa and THRb expression were detected (54, 56). Interestingly, *in vitro* analyses of bone marrow-derived macrophages (BMDMs) in a murine atherosclerosis model suggested that THs can activate both canonical and noncanonical THRa signalling in macrophages. Moreover, TH action via integrin αVβ3 was shown by *in vitro* stimulation of RAW264.7 cells. Here, PI3K or ERK inhibitors as well as tetraiodothyroacetic acid (Tetrac) interfered with TH signalling in RAW264.7 macrophages (57, 58).

**Table 1:** Expression pattern of proteins involved in local TH action in immune cells.

Cell type	THT	DIO	THR	NCS	TSH receptor	Reference
Neutrophil	MCT8, MCT10 (h)	DIO3, DIO1 (h)	THRa (h)	Yes		(5, 69, 70)
Macrophage	MCT8, MCT10, LAT2, OATP4a1	DIO2	THRa >> THRb	Yes		(54, 55, 56, 57, 58)
Monocyte		DIO2 (h)		Yes	Yes	(54, 81, 108)
Dendritic cell	MCT10 >> LAT2	DIO3 >> DIO2	THRa, THRb		Yes	(6, 65, 110)
Natural killer cell	MCT8 (h), MCT10 (h)		THRa (h), THRb (h)		Yes	(87, 108)
T cell			THRa, THRb	Yes	Yes	(97, 98, 109, 110)
B cell			THRa, THRb	Yes	Yes	(104, 108)

If not indicated data are from mice.

DIO, deiodinase; h, human; NCS, noncanonical signaling; THR, TH receptor; THT, TH transporter; TSH, thyroid stimulating hormone.

Functionally, THs may drive pro-inflammatory macrophage responses as they triggered phagocytic activity and nitric oxide production in murine and human macrophage cell lines (58). In line with this, DIO2 deficient BMDMs exhibited impaired phagocytosis and bacterial killing *in vitro* (54, 59). Moreover, THs were described to drive M1 polarization of murine BMDMs *in vitro* while limiting M2 polarization (56, 58). Interestingly, THRb mRNA expression was significantly augmented in M2 macrophages compared to M1 or unpolarised macrophages (56), suggesting that the pro-inflammatory polarisation is mediated by THRa signalling. In contrast to the pro-inflammatory action of THs, lack of THs, that is hypothyroidism has been associated with exacerbated inflammatory responses during an unilateral ureteral obstruction (UUO) model of kidney injury and in atherosclerosis in mice. Here, signalling of THs via THRa was described to limit the secretion of pro-inflammatory cytokine by macrophages (57, 60). In line with this, DIO2 deficient mice displayed an increased susceptibility to acute lung injury indicated by elevated inflammatory chemokine and cytokine levels which could be in part reversed by T3 treatment (61). Thus, the current concept of local TH action within macrophages is at best incomplete and conflicting (Fig. 2).

### Dendritic cells

Similar to macrophages, dendritic cells (DCs) can be found in various non-lymphoid and lymphoid tissues and are critically involved in pathogen surveillance and regulation of adaptive immune responses (62). Two main subsets of DCs have been described, classical DCs (cDCs) and plasmacytoid DCs (pDCs). Upon activation, the latter are the main source of type I interferons, which drive antiviral immunity, whereas cDCs are the major type of antigen-presenting cells (APCs), which initiate T cell responses with type 1 cDCs activating CD8+ T cells whereas type 2 cDCs stimulate CD4+ T helper cells (63, 64). For this purpose, cDCs migrate into lymphoid tissues and undergo maturation after recognition of foreign antigens.

Regarding TH action, MCT10 and LAT2 expression on transcript and protein level was found in murine bone marrow-derived DCs (BMDCs). *In vitro* analysis of T3 uptake by BMDCs in presence of THT inhibitors implied that TH transport was mainly mediated by MCT10. Furthermore, murine BMDCs were described to express DIO2 and DIO3 with DIO3 exhibiting higher enzyme activity in BMDCs *in vitro* (6). Moreover, murine BMDCs were depicted to express THRb and to a lesser extent THRa ((65), Table 1).

*In vitro* treatment with T3 but not T4 was described to induce maturation of BMDCs and to increase their ability to stimulate T cells via an augmented expression of costimulatory molecules, such as MHC class II, CD40 and CD80 (6, 65). Accordingly, T3 was suggested to direct BMDCs towards a pro-inflammatory phenotype driving Il17 mediated immune responses as well as cytotoxic T cell responses in a murine B16 melanoma model (66, 67). Moreover, T3 was shown to reduce the expression of inhibitory molecules such as PD-L1 on BMDCs *in vitro* and thus impair their ability to induce regulatory T cells (Treg) (66). Additionally, *in vivo* tracking of fluorescently labelled BMDCs revealed a stimulatory effect of T3 on DC migration. In line with this, *in vitro* stimulation of BMDCs with T3 increased the expression of migratory marker CCR7 (67). Hence, these studies suggest that T3 may act as a stimulatory regulator of pro-inflammatory responses of DCs *in vitro* (Fig. 2). Yet, gene expression and functional analysis performed in BMDCs only provide insights into the action of cDCs, leaving out the impact of THs on pDCs. Thus, so far nothing is known about the local action of THs in pDCs.

### Neutrophils

During infection, neutrophils are the first cells to be recruited to the site of inflammation and facilitate pathogen clearance via intracellular and extracellular killing. Among others, intracellular killing relies on the phagocytosis of pathogens and the generation of ROS, whereas extracellular killing is exerted by the formation of so-called neutrophil extracellular traps (68).

Neutrophils were the first immune cells in which conversion of T4 into T3 was shown (69). Later studies revealed that MCT8 and DIO3 are expressed in murine neutrophils, whereas MCT10, DIO3, DIO1 and THRa expression was demonstrated in human circulating neutrophils by conventional PCR ((5, 70), Table 1).

Regarding the functional role of THs in neutrophils, DIO3 was shown to be essential for optimal neutrophil activity, in particular for neutrophil migration and intracellular killing mediated by ROS during bacterial infection in mice, suggesting that neutrophils function well when intracellular T3 levels are low ((71, 72), Fig. 2). Moreover, a positive correlation between ROS generation via NADPH oxidase and subclinical hypothyroidism has been reported in humans suggesting an inhibitory TH effect on neutrophil function (7). However, it is still unknown whether the impact of DIO3 on ROS production depends on TH action or is a TH-independent effect. For example, Boelen *et al.*suggested that DIO3 may serve as a source of inorganic iodide required for myeloperoxidase function in oxidative stress supporting a TH-independent effect (73). Besides, human neutrophils were described to exhibit elevated NADPH activity and ROS generation upon stimulation with T3 (8, 74). Interestingly, neutrophils obtained from a patient with a mutation in THRa that lead to TH resistance did not display any functional restraints regarding antibacterial activity, ROS production or migration (75). However, reduced numbers of neutrophilic granulocytes were detected in THRa^-/-^ mice (76). Regulatory effects of THs on ROS production in human neutrophils were suggested to be mediated by noncanonical effects via a membrane-bound target of THs, i.e. integrin αVβ3 (77), but a comprehensive concept linking TH transport, metabolism and signalling in neutrophils and conclusion for neutrophil function is still missing.

### Monocytes

Following neutrophils, monocytes migrate to the site of infection, where they release pro-inflammatory cytokines and may differentiate into macrophages or DCs (78). Of note, monocyte-derived macrophages and DCs differ markedly from their tissue-resident counterparts since they display a more pronounced inflammatory phenotype and thus are commonly associated with more severe immunopathology (79, 80).

TH action in monocytes is rarely studied so far and most research focused on monocyte-derived macrophages. The human monocyte cell line THP-1 was shown to express DIO2 via qRT-PCR and high amounts of integrin αVβ3 ((54, 81), Table 1). Functionally, THs were described to enhance the antibacterial immune response of monocyte-derived macrophages indicated by enhanced phagocytic activity as well as elevated expression of inducible nitric oxide synthase (iNOS) in these cells (58). THs were found to limit the differentiation of monocytes into macrophages *in vitro* while promoting the differentiation into DCs (56, 82). Furthermore, *in vitro* stimulation of THP-1 monocytes with THs impaired their migration in response to several chemoattractant proteins (83). In line with this, elevated frequencies of monocyte-derived macrophages were found during LPS-induced endotoxemia in hypothyroid mice and T3 application restored normal macrophage frequencies (56). Moreover, inflammatory responses of THP-1-derived macrophages to silica were attenuated in presence of THs *in vitro* ((84), Fig. 2). Thus, little is known about the impact of THs on monocytes whereas the current understanding of local TH action in monocyte-derived macrophages is conflicting.

### Natural killer cells

Natural killer (NK) cells represent a unique group of innate lymphocytes within the immune system (85). Upon infection, NK cells are readily recruited to the site of inflammation to lyse infected cells. Activation of NK cells is regulated by the balance of inhibitory and activating receptors which interact with their counterparts expressed on host cells (86).

Regarding local TH action, human uterine NK cells were suggested to express THTs MCT8 and MCT10, as well as both types of THRs based on immunocytochemical analysis ((87), Table 1). However, the functional role of TH signalling within NK cells is controversial (Fig. 2). Exogenous T3 was described to augment NK cell activity of older individuals with low serum T3 levels *in vitro*, whereas hyperthyroidism due to Graves’ disease was proposed to limit NK cell activation (88, 89). Similarly, systemic treatment of old mice with T4 increased NK cell activity and sensitivity to IFNγ, while hyperthyroid mice displayed impaired NK cytotoxic function (90, 91).

## Local action of THs in adaptive immune cells

### T cells

Cellular immune responses of the adaptive immune system are mediated by T lymphocytes, which can be divided into CD4+ and CD8+ T cells (92). Upon activation in lymphoid tissues by DCs, CD8+ T cells migrate to the site of infection to clear infected cells via the induction of apoptosis, whereas CD4+ T cells differentiate into various types of T helper cell subsets supporting distinct cellular immune responses (93, 94). Importantly, following pathogen clearance a small fraction of effector T cells differentiate into memory T cells accelerating immune responses upon reinfection (95, 96).

So far little is known about the expression of proteins involved in local TH action in T lymphocytes (Table 1). THRa and THRb were described to be expressed by T cells on mRNA and protein levels, respectively (97). Moreover, TH deiodination was observed within human lymphocytes, but the expression or activity of DIOs in T cells has not been addressed yet (98). Furthermore, analyses of TH transport into T cells are still missing.

Different indirect effects of THs on T cell function were described, which are mediated via the adjustment of DC responses (see above). Yet, THs were also suggested to directly modulate T cell proliferation and activation as *in vivo* T4 application enhanced T cell numbers and anti-tumour immunity in a murine lymphoma model (97, 99). Accordingly, reduced tumour rejection was observed in murine lymphoma model under chronic stress, which is associated with reduced systemic TH levels (100). On the contrary, *in vitro* stimulation of human T lymphocyte cell lines as well as peripheral blood lymphocytes with THs were described to increase T cell apoptosis (101), implying a negative role of THs on T cell immunity. Thus, our current knowledge on the role of THs in T cells in general, in anti-tumour immunity, and in T cell subsets, is incomplete and contradictory (Fig. 2).

### B cells

Humoral immune responses of the adaptive immune compartment are facilitated by B lymphocytes, which can secrete different types of immunoglobulins (Ig) (102). Activation of B cells, based on the recognition and affinity of foreign antigens to the B cell receptor (BCR), initiates activation and differentiation of B cells into short-lived plasmablasts or long-lived plasma cells and memory B cells in germinal centres (103).

Similar to T cells, only THRa and THRb were found to be expressed in B cells ((104), Table 1), whereas other components of local TH action have not been identified yet. Nevertheless, THs were proposed to be essential for primary B cell development since reduced numbers of pro-B cells and pro-B cell proliferation was found in absence of THRa signalling in a murine model of THRa resistance (76, 104, 105). In line with this, accelerated B cell proliferation was observed upon *in vitro* stimulation of human peripheral blood B lymphocytes with T3 (106). Thus, THs and THRa might be important for proper B cell immune responses, but further studies are required to study the direct and indirect actions of THs in the development, activation and differentiation of B cells (Fig. 2).

## TSH within the immune system

Although still a matter of debate, TSH itself has been proposed as a potential regulator of the immune system as different immune cells were shown to express TSHR (for review see (107)). In agreement with this, recombinant human TSH was shown to promote lymphocyte proliferation and activation, including NK, T and B cells in thyroidectomised patients *in vivo*(108). Moreover, TSH was suggested to promote thymic T cell development in humans and mice, based on the interaction with thymic T cell expressed TSHR (109). Furthermore, the phagocytic activity and pro-inflammatory cytokine secretion of DCs were enhanced in presence of TSH *in vitro*(110). Additionally, lymphocytes and splenic DCs were suggested to express TSH with DCs being the primary source. Thus, TSH has been proposed to act as a humoral mediator similar to cytokines within the immune system. Interestingly, murine myeloid progenitor cells in the bone marrow produce a specific splice variant, TSHb, which might indicate a specific feature of immune cell-derived TSH (111). Based on the finding of synthesis of TSH within the immune system, immune cells were implied to be involved in the restoration of TH homeostasis during recovery of NTIS (107). However, evidence for a restoring function of the immune system via TSH on systemic TH levels during NTIS has yet to be provided.

## The unknowns of TH action on immune responses

Although many studies have been performed to understand the interplay of THs and the immune system, the results so far only represent puzzle pieces with many unclear aspects, which comprise three major unknowns: (i) the components mediating TH action; (ii) the impact of THs on immune cell function; (iii) tissue-specific TH availability in health and disease.

### Components of local TH action

The exact components mediating local TH action within distinct immune cells are largely unknown so far due to several limitations of previous studies. Firstly, many data are based on mRNA analysis whereas the knowledge on the ‘functional’ expression of proteins involved in local TH action within immune cells is fragmentary, in part due to lack of specific antibodies, for example, for THRa and THRb (Table 1). Second, a comprehensive analysis on the expression of THTs, DIOs and THRs within distinct immune cell populations is lacking. For example, THTs involved in TH uptake into T or B cells are unknown and in-depth analyses of TH action in subpopulations of immune cells, such as CD4+ and CD8+ T cells, tissue-resident and monocyte-derived macrophages, or cDC and pDC subsets, are still missing (Table 1). Third, previous analyses were mainly performed in cell lines as well as bone marrow-derived cells, and thus, obtained results should be viewed with caution. Expression levels in cell lines may differ from primary immune cells and, similarly, prolonged ex vivo culture of progenitor cells might cause alterations in the expression of proteins involved in local TH action, which are among others controlled in an autoregulatory fashion by THs (25, 26, 112). Fourth, although the expression of proteins involved in local TH action was studied partly in mice and partly in humans, profound comparative analyses of the two species to ensure translational relevance are largely lacking. Yet, different expression patterns of THTs and DIOs were observed, for example, in murine and human neutrophils (5, 70), potentially indicating species-specific differences.

### Impact of THs on immunity

The precise effect of local TH action on the function of individual immune cells is poorly understood. First, current results on the functional aspects of THs within the immune system are controversial and suggest stimulatory as well as inhibitory effects of THs within the same immune cell population (Fig. 2). For example, THs were described to drive M1 polarization of macrophages* in vitro*, whereas THs were shown to limit inflammatory macrophage responses during an UUO model of kidney injury (56, 60). Likewise, THs were suggested on the one hand to enhance anti-tumour immunity and on the other hand, to trigger T cell apoptosis (99, 101). Second, similar to expression analysis, no in-depth analysis of specific immune cell subtypes (e.g. CD4+ T cells, CD8+ T cells) has been performed in this context. Likewise, little is known about the impact of activation and polarisation on the role of THs in immune cell function, although it has been noted, that activation and polarisation of, for example, macrophages alters the expression of proteins involved in local TH action. Here, stimulation of BMDMs with LPS increased the expression of DIO2 and THRa (54), while an augmented expression of THRb was found in M2 macrophages compared to unpolarised or M1 macrophages (56). Thus, the functional outcome of TH stimulation might differ in activated immune cells between distinct subtypes, such as T helper 1 and regulatory CD4+ T cells as well as CD8+ T cells. Accordingly, the pathological models used to characterise the impact of THs on the immune system might have gained different and contrasting results, depending on the immune cell populations activated within the distinct models.

### Local TH availability

Our current knowledge about local mechanisms controlling TH tissue concentrations in health and disease and their influence on proper immune responses is limited. For example, the TH status of lymphoid tissues, such as lymph nodes and spleen, as well as distinct peripheral tissue, such as lung, liver or gut, is largely undefined both in health and disease. Interestingly, increased expression of DIO2 was observed in a murine model of ventilator-induced lung injury (VILI) (61, 113), whereas augmented expression of DIO3 has been described under hypoxia, a hallmark of, for example, healthy intestinal but also inflamed tissues (114). Thus, local TH availability to resident immune cells might differ and be an additional factor involved in immune tolerance and development of immune cells at a steady state. Changes in TH environment due to immune cell migration or specific alterations in TH tissue concentrations upon infection/inflammation may be involved in shaping immune responses leading to protective or detrimental adaptions. Of note, although T3 is in general considered as the active form of TH, other TH metabolites such as rT3 and 3,5’ T2 have been recently proposed to regulate cellular functions as well (15). Thus, the role of THs in immune responses may depend on the affected tissue and therefore might explain controversial results obtained by several pathological models so far.

A better understanding of local TH action within immune cells and its impact on immune cell function will be crucial to infer the therapeutic potential of TH interventions during different types of diseases. Additionally, extending our knowledge of local TH environments might help to define the clinical relevance as well as being aware of critical issues to optimize the therapeutic benefit.

## Future implications

Further studies are required to provide a more detailed view on the local action of THs in innate and adaptive immune cells. These studies should address the expression of components involved in local TH action in immune cells, taking the effect of activation as well as differentiation into account. Likewise, in-depth analysis regarding the impact of TH signalling on the function of distinct immune cell populations such as naïve and effector cells may be performed. Here, basic *in vitro* experiments as well as *in vivo* studies might shed light on the role of THs in different stages of immune responses and their mode of action. Consideration of different pathological models such as acute and chronic viral infections, but also inflammatory diseases such as non-alcoholic steatohepatitis (NASH), acute on chronic liver failure, stroke, or myocardial infarction might extend our knowledge on the impact of THs on immune cell function and may allow clinical implications for TH modulation in different diseases.

## Conclusion

In recent years, there is growing evidence for a direct influence of THs on the immune system. Cells of both, the innate and adaptive immune systems, express a variety of components involved in local TH action and are sensitive to THs affecting immune cell function. Yet, further studies are necessary to define the impact of THs on different immune cells in more detail and to address the clinical implications.

## Declaration of interest

The authors declare that there is no conflict of interest that could be perceived as prejudicing the impartiality of this review.

## Funding

This work was funded by the Deutsche Forschungsgemeinschaft (DFG, German Research Foundation): Project-ID 424957847 – TRR 296; RTG 1949.
